# *PIK3CG* single nucleotide polymorphisms are associated with poor responsiveness to clopidogrel and increased risk of ischemia in patients with coronary heart disease

**DOI:** 10.1097/MD.0000000000007566

**Published:** 2017-09-08

**Authors:** Ke-Cheng Li, Shu-Hong Yu, Bao-Zhong Zhuge

**Affiliations:** a Department of Clinical Laboratory, People's Hospital of Rongcheng, Rongcheng; b Department of Blood Transfusion, Yantai Yuhuangding Hospital, Yantai; c Department of Clinical Laboratory, Linyi People's Hospital, Linyi, P.R. China.

**Keywords:** antiplatelet therapy, clopidogrel, coronary heart disease, gene polymorphism, haplotype, percutaneous coronary intervention, *PIK3CG*

## Abstract

**Background:**

This study explores the associations between *PIK3CG* single nucleotide polymorphisms (SNPs, rs1129293 and rs17398575) and patient responsiveness to clopidogrel to evaluate the risks of ischemia in patients with coronary heart disease (CHD).

**Methods:**

The study consisted of 513 CHD patients who received clopidogrel as part of antiplatelet therapy, after percutaneous coronary intervention. According to the patient responsiveness to clopidogrel, the subjects were assigned to either clopidogrel-resistant (CR) or clopidogrel-sensitive (CS) groups. CR group was determined by patients’ platelet aggregation rate of ≥70% and poor responsiveness to clopidogrel, and CS group by patients’ platelet aggregation rates of <70% and good responsiveness to clopidogrel. Polymerase chain reaction using TaqMan probe was employed to detect *PIK3CG* polymorphism. Haplotype and linkage disequilibrium analyses were performed. Prognosis analysis was performed using the Kaplan-Meier curve.

**Results:**

Significant difference was found in genotype and rs1129293 and rs17398575 allele frequency between the CR and CS groups. Haplotype analysis indicated that the frequency of TG allele was higher in the CR group compared with the CS group, and the frequency of CA allele was lower in the CR group compared with the CS group. Patients with rs1129293 CT + TT genotype and T allele, rs1129293 AG + GG genotype and G allele exhibited an increased CR risk. Logistic regression analysis determined hypertension history as an independent risk factor for CR. The Kaplan-Meier curve suggests that distribution curve of cumulative probability nonischemic events was different between patients with rs1129293 and rs17398575 alleles. Stable CHD patients with TT genotype of rs1129293 allele and GG genotype of rs17398575 allele showed poorer prognosis compared to those with other genotypes and patients with acute coronary syndromes.

**Conclusion:**

A positive correlation may exist between *PIK3CG* SNPs (rs1129293 and rs17398575) and patients with poor responsiveness to clopidogrel. These findings show that this factor may contribute to an increased risk of ischemia in patients suffering from CHD.

## 1 Introduction

Coronary heart disease (CHD) is a term used to describe a group of diseases that is characterized by a narrowing of the blood vessels that supply blood and oxygen to the heart. A lot of factors that have been correlated and associated with the onset of CHD include high blood pressure, smoking, diabetes, lack of exercise, obesity, poor diet, high blood cholesterol, and high blood sugar. Dual antiplatelet therapy (DAT) with aspirin and clopidogrel is a commonly recommended treatment used to prevent adverse cardiovascular events in patients suffering from CHD after stent implantation.^[1,2]^ Unfortunately, this treatment regimen still fails to produce good antiplatelet effects in some patients, leading to further ischemic complications such as stent thrombosis, cardiovascular death, nonfatal myocardial infarction, and strokes.^[3,4]^ Research has shown that these inconsistencies in complications, although using the same treatment regimen, could be partially linked to toxicities that arise in different individuals’ response to drugs.^[5]^ Thus far, the exact mechanism of the interindividual variability has not yet been fully elucidated. In addition to clinical factors such as age, renal failure, obesity, diabetes, high plasma fibrinogen, and lack of adherence,^[6–8]^ accumulating evidence suggest that genetic polymorphism is closely associated with patient's response to clopidogrel.^[9,10]^

Phosphatidylinositol-4,5-bisphosphate 3-kinase, catalytic subunit gamma (*PIK3CG)*, is a gene responsible for encoding the protein called PI3Kγ or p110γ which is located at the chromosome 7q223.^[11]^ P110γ is part of the PI3K family of enzymes that is involved in cellular functions such as cell proliferation, differentiation, and intracellular trafficking. Studies have suggested that PI3Kγ plays an important role in the development of cardiovascular diseases such as atherosclerosis, myocardial infarction, and thrombosis.^[12,13]^ Its expression has also been detected in several cardiac cells including cardiomyocytes, vascular endothelial cells and smooth muscle cells, and platelets, thus establishing a link between its expression and various diseases.^[14,15]^ P110γ expression is typically restricted and predominantly detected in leukocytes.^[16]^

In the G-protein-couple receptors (GPCRs) signaling pathway, PI3Kγ is a downstream isoform activated by GPCRs through the β/γ complex, which binds to heterotrimetric G protein subunitβ/γ and GPCRs.^[17]^ A retrospective review suggests that GPCRs/PI34Kγ/PIP3 signaling pathway controls a variety of immune modulatory and vascular functions, including respiratory bursts, cell recruitment, mast cell reactivity, platelet aggregation, endothelial activation, and smooth muscle contractibility.^[18]^ Recently, a genome-wide meta-analysis has identified *PIK3CG* as a loci associated with platelet aggregation.^[19]^ With this knowledge, we hypothesize that *PIK3CG* polymorphism may be a potential indicator to predict the antiplatelet effect of clopidogrel in patients suffering from CHD.

In the present study, we aim to investigate the role of *PIK3CG* polymorphism in interindividual responses to clopidogrel. The study will be conducted by determining the different platelet aggregation rate in patients with 2 *PIK3CG* singly nucleotide polymorphisms (rs1129293 and rs17398575) and the occurrence of ischemic events by the means of an 18-month follow-up.

## 2 Materials and methods

### 2.1 Ethics statement

This research was conducted according to the protocols regulations established by the Linyi People's Hospital. All the experiments conducted in this study were in accordance with the Declaration of Helsinki.^[20]^ Informed consents were obtained from all the subjects and their family members. This investigation was approved by the local institutional review board.

### 2.2 Study subjects selection

A total of 513 diagnosed CHD patients were hospitalized in the Linyi People's Hospital between December 2012 and November 2014. All patients were diagnosed with coronary atherosclerotic heart disease^[21]^ and were currently receiving antiplatelet therapy with clopidogrel following a percutaneous coronary intervention (PCI) procedure. The exclusion criteria for this study were as follows: patients treated with cilostazol and IIb/IIIa receptor antagonists simultaneously, patients treated with calcium-ion antagonists, patients allergic to clopidogrel or showing contraindications to clopidogrel, patients suffering from hematological system diseases and deemed high-risk for bleeding and hemorrhage, patients with malignant tumors, patients who were pregnant, and patients suffering from severe hepatic and renal dysfunction. According to responsiveness to clopidogrel and their platelet aggregation rates, patients were assigned to 2 study groups: clopidogrel-resistant (CR) (platelet aggregation rates ≥70% with poor responsiveness to clopidogrel) and clopidogrel-sensitive (CS) (platelet aggregation rates <70% with favorable responsiveness to clopidogrel).

### 2.3 Data collection

A questionnaire survey was conducted to collect data from all patients participating in this study. The survey included demographic data such as name, gender, age, height, and contact information. Patient's medical history (particularly in regard to hypertension, diabetes mellitus, hyperlipidemia, and stroke), medicines (statins, β receptors blocker, and other medicines) used and personal habits (smoking and drinking history) were also included.

### 2.4 Coronary angiogram, percutaneous coronary intervention, and antiplatelet therapy

All patients participating in this study have previously received conventional coronary angiograms before undergoing conventional PCI. Their results were determined in accordance with the American College of Cardiology/American Heart Association guidelines.^[22]^ The operation was deemed successful if it fulfilled the following criteria: postoperative stenosis was <20%; thrombolysis in myocardial infarction (TIMI) was reestablished to normal flow which completely fills the distal coronary bed; and no serious implications were observed. The operation was seen as a clinical success when patients’ symptoms and signs of myocardial ischemia disappeared. Antiplatelet therapy was given to the patients according to the following regimen: Patients were given 300 mg of clopidogrel 24 hours before the surgery and 75 mg after the operation.

### 2.5 Detection of patient platelet aggregation rate

Blood samples, 5 mL each, were collected from the median cubital vein of patients who underwent antiplatelet therapy for at least 7 days (5 mL/person). Collected blood samples were transferred to an anticoagulative tube containing 3.8% sodium citrate (J&K Scientific, Peking, China), and thoroughly mixed and detected within a 2-hour duration to avoid damage to the blood contents. Turbidimetric platelet aggregometry (TPA) was applied to measure the platelet aggregation rate. Autologous platelet-rich plasma (PRP) and platelet-poor plasma (PPP) were prepared by centrifugation. According to the adenosine diphosphate (ADP) concentration difference (2 μmol/L) between PRP and PPP, platelet aggregation rate was determined using Optical principles. For post-clopidogrel therapy, the 2 μmol/L ADP-platelet aggregation rate was categorized as ≥70%. Patients who had aggregation rates of >70% were considered as CR. In contrast, patients who had platelet aggregation rates of <70% were categorized as CS.^[23]^

### 2.6 DNA extraction and gene identification

Genomic DNA was extracted from anticoagulated whole blood sample in accordance with the instructions of the DNA extraction kit provided by QIAGEN GmbH, Hilden, Germany. Ultraviolet spectrometry set at 260/280 nm was used to detect optical density and for the measurement of DNA concentration. Two SNPs, rs1129293 and rs17398575, were selected. The primers of polymerase chain reaction (PCR) (Table 1) amplification were designed with the help of Primer Premier 5.0 (Premier Biosoft International, Palo Alto, CA) and synthesized by the Shanghai Sangon Biological Engineering Technology and Services Co. Ltd (Shanghai, China). The TaqMan probe was adopted for SNP sequencing. The PCR contents comprised a total of 25 μL of the following components: 1.25 μL of TaqMan probe, 12.5 μL of 2 × Power Taq MasterMix (including dNTPs, TaqDNA polymerase, MgCl_2_, dye, reaction buffer, PCR enhancer, optimizers, and stabilizer), 11.25 μL of DNA stoste, and double-distilled water. The PCRs were set according to the following conditions: 95°C predegeneration for 3 min, 95°C degeneration for 15 min, 60°C annealing for 1 min; the PCR was set to repeat for a total of 45 cycles. TaqMan probe was applied to detect the polymorphism in rs1129293 and rs17398575 SNPs. According to the fluorescence curve produced beforehand, real-time fluorescence quantification PCR machine was produced that reflected the (S1000, Bio-Rad, Inc., Hercules, CA) rs1129293 SNPs including CC, CT, and TT, and rs17398575 SNPs including AA, AG, and GG.

**Table 1:**
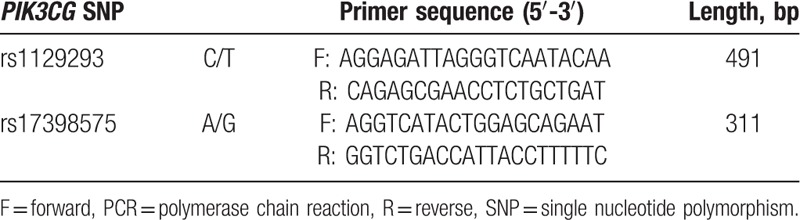
Primer sequences for PCR amplification of *PIK3CG* rs1129293 and rs17398575 polymorphisms.

### 2.7 Follow-up

An 18-month follow-up was conducted to record and keep track of any ischemic occurrences in the patients who partook in the study. These follow-ups were done through phone call and clinical visits. The conditions that we classified as an ischemic occurrence included cardiac death, nonfatal myocardial infarction, and ischemic stroke. The follow-up criteria included the number of cardiac deaths, which was divided into those caused by cardiac diseases and those caused by unknown etiologies; ischemic stroke caused by cerebral ischemia and confirmed by imaging evidence; and nonfatal myocardial infarction diagnosis—increased cTnT (more than 1 instance) over the upper limit of 95% reference range (0.03 ng/mL) along with at least 1 of the following phenomena: clinical symptoms of myocardial ischemia, new changes in myocardial ischemia shown by electrocardiogram, pathological Q waves detected in electrocardiogram, and newly emerged loss in myocardial viability and regional wall motion abnormalities on image maps.

### 2.8 Statistical analysis

Statistical analysis was performed using SPSS 21.0 software for data analysis (SPSS Inc, Chicago, IL). Comparisons between groups of general information were conducted by *t* and chi-square test (χ^2^). Genotype distribution was detected according to the Hardy-Weinberg equilibrium, and χ^2^ test was employed to help determine SNPs genotype distribution and allele frequency. SHEsis software was used for haplotype analysis. The association between genotype and prognosis of CHD patients was analyzed using a Kaplan-Meier curve. The risk of clopidogrel-resistance in patients was evaluated with a logistics regression analysis. *P* <.05 was considered as statistically significant.

## 3 Results

### 3.1 Comparison of baseline characteristics between the clopidogrel-resistant and clopidogrel-sensitive groups

Patients were divided into CR (n = 101) and CS (n = 412) groups, depending on how well they responded to clopidogrel. *t* Test showed that the differences in the treatment of patients with hypertension using ACEi, ARB, and proton pump inhibitors between CR and CS groups were significant (all *P* <.05). The 2 groups showed no significant differences in their age, gender proportion, body mass index (BMI), history of smoking, drinking, stroke, hyperlipidemia, diabetes, and usage of statins and β receptor blockers (all *P* >.05) (Table 2).

**Table 2:**
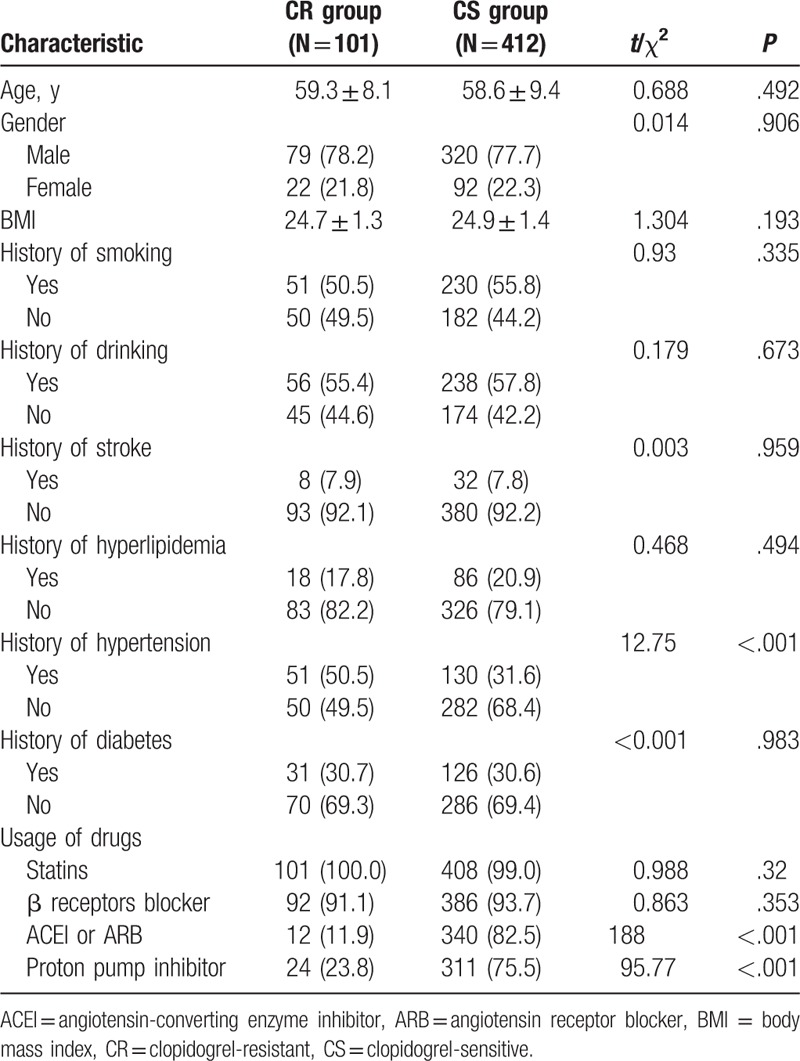
General characteristics of the included patients in CR and CS groups.

### 3.2 PCR results

**Figure 1:**
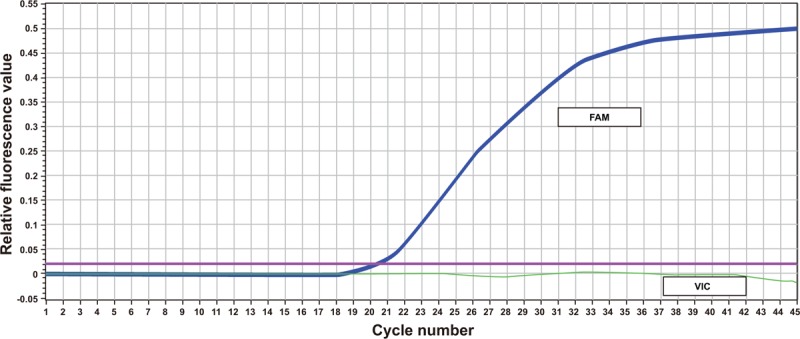
PCR curve of TaqMan probe for homozygotes. PCR = polymerase chain reaction, SNP rs17398575: GG genotype; SNP rs1129293: TT genotype.

**Figure 2:**
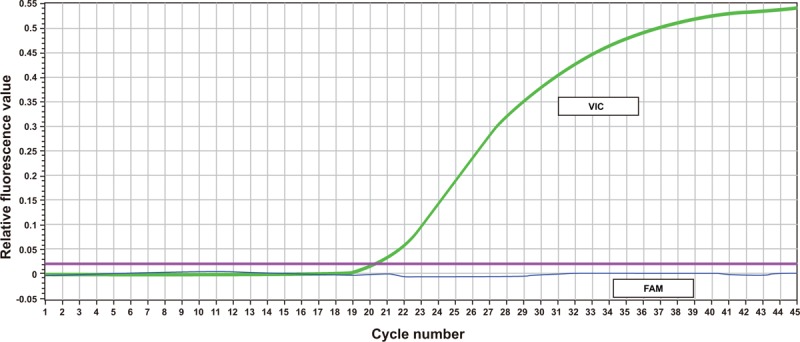
PCR curve of TaqMan probe for homozygotes. PCR = polymerase chain reaction; SNP rs17398575: AA genotype; SNP rs1129293: CC genotype.

**Figure 3:**
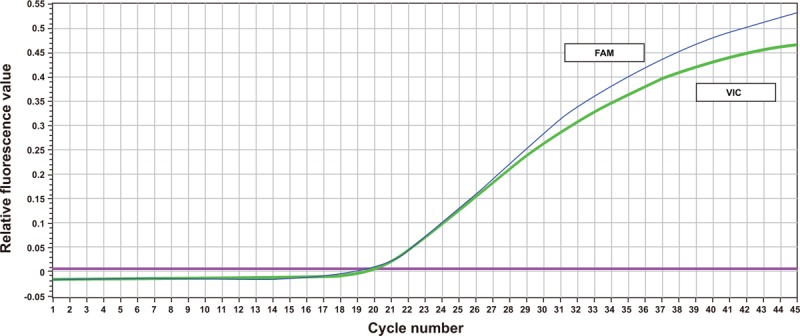
PCR curve of TaqMan probe for heterozygosis. PCR = polymerase chain reaction; SNP rs17398575: AG genotype; SNP rs1129293: CT genotype.

After TaqMan-probe PCR, the DNA samples were amplified into 3 different curves. T allele in *rs112929* gene was marked by 6-carboxyfluorescein (FAM), and G allele in rs112929 was marked by green fluoresent protein of Victoria (VIC). After PCR amplification, 3 different results (CC, CT, and TT) were presented. G allele in the *rs112929* gene was marked by FAM, and A allele in *rs112929* was marked by VIC. After PCR amplification, 3 different results (AA, AG, and GG) were presented (Figs. 1–3).

### 3.3 Hardy-Weinberg equilibrium and genotype distribution of *PIK3CG*

As shown in Table 3, genotype distribution of *rs1129293* and *rs17398575* genes was in accordance with Hardy-Weinberg equilibrium (*P* > .05), suggesting that selected patients were representative of the population (Table 3).

**Table 3:**
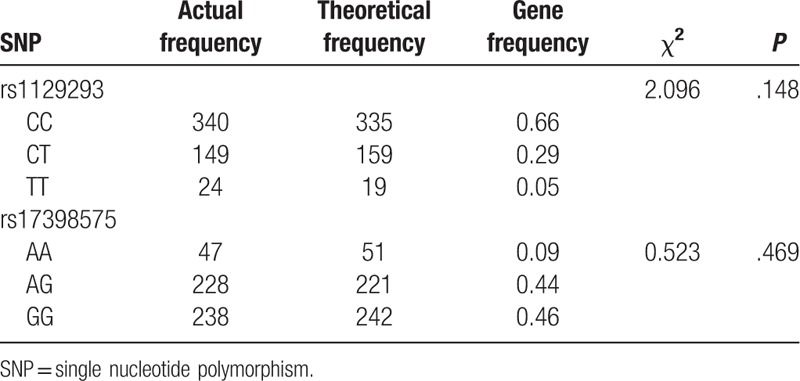
The results of Hardy-Weinberg equilibrium among all patients.

Genotypic and allele frequencies of *rs1129293* and *rs17398575* genes presented significant differences between CR and CS groups (all *P* <.05). Patients with rs1129293 CT + TT genotype were 2.45 times more susceptible to CR than the patients with rs1129293 CC genotype (CT + TT vs CC: OR = 2.425, 95% CI = 1.556–3.779). Additionally, patients with rs1129293 T allele were 2.334 times more susceptible to CR than the patients with rs1129293 C allele (T allele vs C allele: OR = 2.334, 95% CI = 1.644–3.314). Patients with rs17398575 AG + GG genotype were more susceptible to CR with a 6.069 times higher risk of CR than the patients with rs17398575 AA genotype (AG + GG vs AA: OR = 6.069, 95% CI = 1.447–25.460). Moreover, patients with rs17398575 G allele were more susceptible to CR with a 2.270 times higher risk of CR than the patients with rs17398575 A allele (G allele vs A allele: OR = 2.270, 95% CI = 1.551–3.323) (Table 4).

**Table 4:**
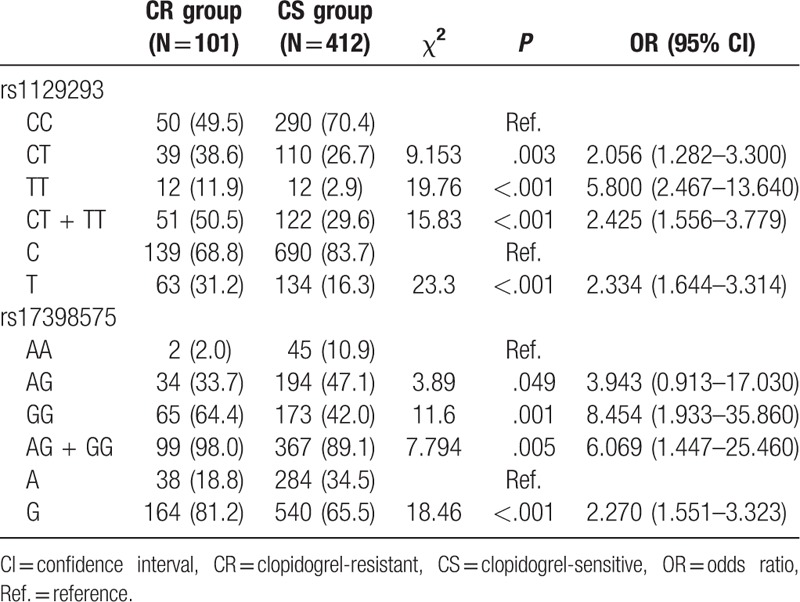
Genotype and allele frequency of *PIK3CG* rs1129293 and rs17398575 polymorphisms of CR and CS groups.

### 3.4 Haplotyping analysis of PIKCG SNPs

Linkage disequilibrium analysis and haplotype analysis in *PIKCG* SNPs (rs1129293 and rs17398575) was performed using the SHEsis software. Haplotyping results revealed that the TG frequency was higher in the CR group compared with the CS group, whereas CA frequency was lower in the CR group compared with the CS group (all *P* <.05) (Table 5).

**Table 5:**
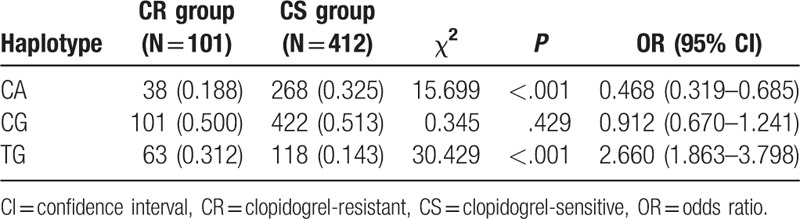
Haplotype analysis of *PIK3CG* rs1129293 and rs17398575 polymorphisms of CR and CS groups.

### 3.5 Result of logistic regression analysis

Logistic regression analysis was conducted using CR as the dependent variable, and hypertension history, usage of ACEI or ARB, proton pump inhibitor, and genotype frequency of rs1129293 and rs17398575 as independent variables. Table 6 presents the results of logistic regression analysis. Hypertension history was an independent risk factor for CR (OR = 1.989, 95% CI = 1.0361–3.820; OR = 2.563, 95% CI = 1.341–4.899; OR = 2.942, 95% CI = 1.439–6.016, all *P* <.05).

**Table 6:**
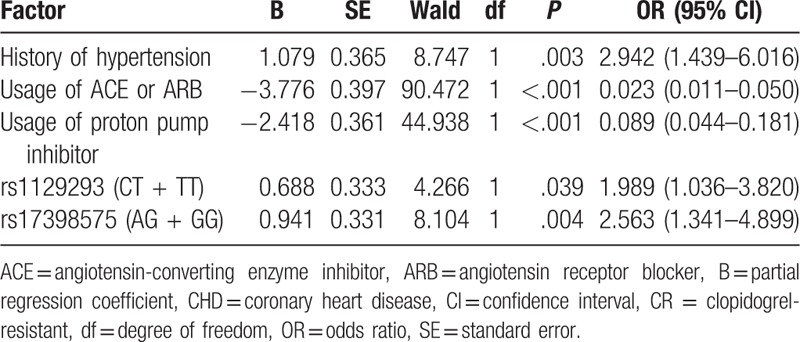
Logistic regression analysis to screen the risk factors for CR in CHD patients after antiplatelet therapy of clopidogrel.

### 3.6 Association between *PIK3CG* polymorphism and prognosis

**Figure 4:**
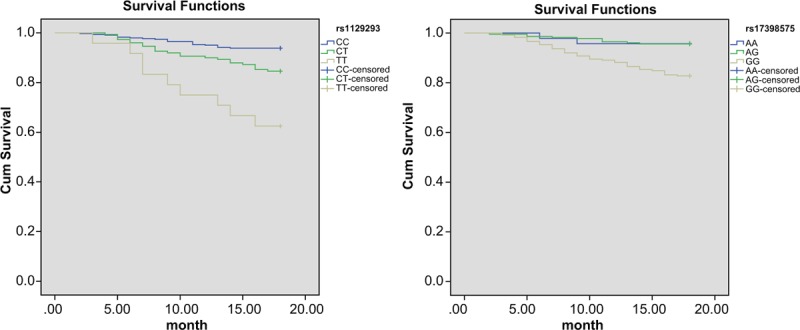
The Kaplan-Meier curve of ischemic events of CHD patients with rs1129293 (A) and rs17398575 (B). CHD = coronary heart disease.

All patients, who participate in our study, completed the requirement of 18-month follow-up procedure. Among these 513 patients, 53 presented with the aforementioned ischemic issues, including 15 cardiac deaths, 27 cases of nonfatal myocardial infarctions, and 12 cases of ischemic strokes. Significant differences were found in the genotype frequency of rs1129293 and rs17398575 (*P* < .05) between the ischemic event group and the ischemic event-free group (Table 7). Kaplan-Meier curve revealed significant differences in the distribution curve of cumulative probability of nonischemic events in patients with *rs1129293* and *rs17398575* genes (all *P* <.05) (Fig. 4).

**Table 7:**
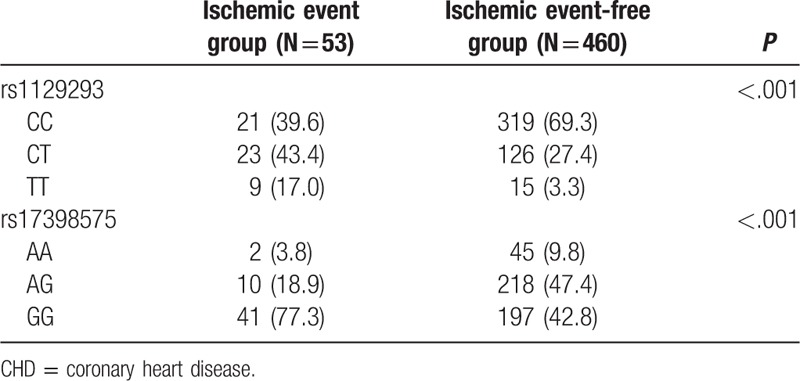
Associations between prognosis of CHD and *PIK3CG* rs1129293 and rs17398575 polymorphisms.

**Figure 5:**
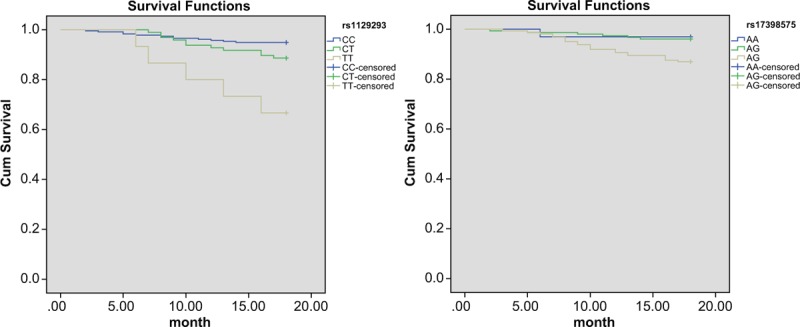
The Kaplan-Meier curve of ischemic events of stable CHD patients with rs1129293 (A) and rs17398575 (B). CHD = coronary heart disease.

Among the 513 patients, 348 patients presented with stable CHD and 165 patients presented with acute coronary syndrome. During the follow-up duration, 28 patients with stable CHD suffered from ischemic diseases: 7 cases of cardiac death, 15 cases of nonfatal myocardial infarction, and 7 cases of cerebral arterial thrombosis. There was remarkable difference in rs1129293 and rs17398575 genotype frequency between the ischemic event groups and the ischemic event-free groups (Table 8). Kaplan-Meier analysis showed that patients with the TT genotype of SNP rs1129293 and GG genotype of SNP rs17398575 presented a poorer prognosis compared with other patients (all *P* <.05) (Fig. 5).

**Table 8:**
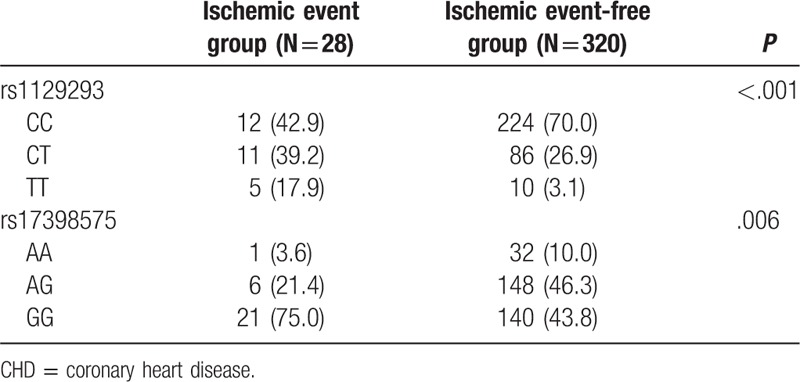
Associations between prognosis of stable CHD and *PIK3CG* rs1129293 and rs17398575 polymorphisms.

**Figure 6:**
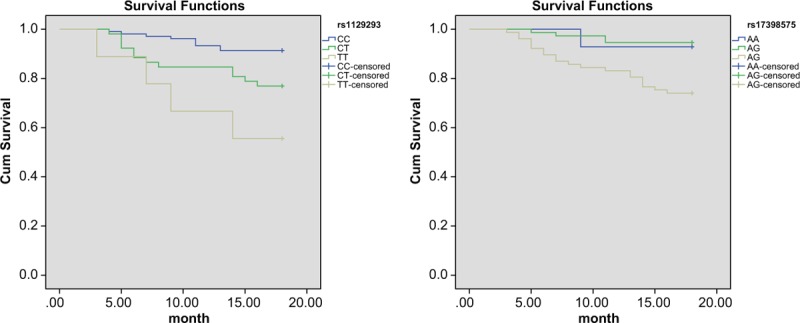
The Kaplan-Meier curve of ischemic events of acute coronary syndrome patients with rs1129293 (A) and rs17398575 (B).

Moreover, among the 165 patients with acute coronary syndrome, 25 cases presented with ischemic occurrences, which included 8 cases of cardiac death, 12 cases of nonfatal myocardial infarction, and 5 cases of cerebral arterial thrombosis. Patients with TT genotype of SNP rs1129293 and GG genotype of SNP rs17398575 showed a higher ischemic occurrence risk compared to patients with other genotypes (all *P* <.05) (Table 9). Kaplan-Meier analysis helped confirm that the aforementioned patients with TT genotype of SNP rs1129293 and GG genotype of SNP rs17398575 also presented a poorer prognosis compared with other patients (all *P* <.05) (Fig. 6).

**Table 9:**
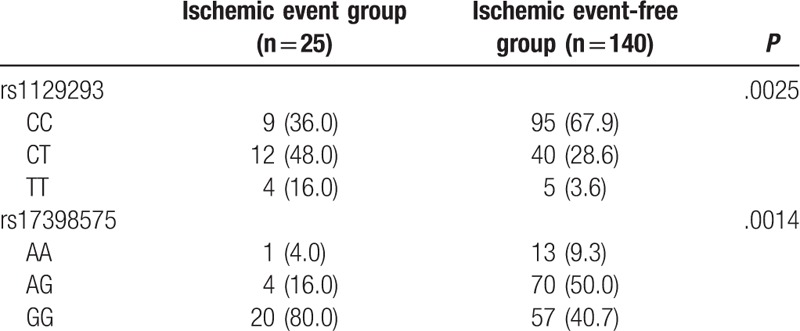
Associations between prognosis of acute coronary syndrome and *PIK3CG* rs1129293 and rs17398575 polymorphisms.

## 4 Discussion

It has been well established that drug response is different in every individual, thus calling for individual drug schemes for better efficacy. A lot of researches have signified the effectiveness of genotyping in predicating individual drug response.^[24,25]^ Evidence also supported correlations between adverse cardiovascular events and interindividual drug response to antiplatelet clopidogrel.^[26,27]^ In this study, we explore the association between *PIK3CG* polymorphisms and the antiplatelet responsiveness of clopidogrel, in hopes of paving a way for novel findings for the evaluation of interindividual drug responsiveness in patients suffering from CHD.

Our findings show there was a significant difference in the genotype and allelic frequencies of *PIK3CG* SNPs (rs1129293 and rs17398575) between CR and CS groups. This suggests that the rs1129293 and rs17398575 SNPs may act as useful biomarkers for the prediction of patient responsiveness to clopidogrel. Furthermore, patients with rs1129293 CT + TT and rs17398575 AG + GG genotypes showed a higher risk of CR than the patients with rs1129293 CC and rs17398575 AA genotype. We could thus interpret that both rs1129293 CT + TT and rs17398575 AG + GG allelic variations could be risk factors for CR. On basis of our results, we suggest that genotyping of the *PIK3CG* gene should be conducted in patients before the administration of clopidogrel.

Upon entering the human body, clopidogrel produces an active role through enzymatic oxidation by cytochrome P450 2C19. This is followed by an inevitable blockade of the combination between ADP and its platelet receptor P_2_Y_12_, thereby allowing the drug to exert its inhibitive role in platelet aggregation.^[28,29]^ Studies have proven that PI3Kγ is activated by Gi-coupled P_2_Y_12_ ADP receptor and PI3Kγ-deficient platelets. Though PI3K responds to thrombin and thromboxane, it showed a reduction in both platelet aggregation and fibrinogen after stimulation with ADP.^[18,30,31]^ We hypothesized that there could be a relationship between *PIK3CG* polymorphisms and interindividual responsiveness to clopidogrel. Based on this hypothesis, we could determin the significance of the *PIK3CG* SNPs (rs1129293 and rs17398575) before drug administration. By conducting a 6-month follow-up, results suggested and found that the occurrence of ischemic events varies with the different genotypes of rs1129293 and rs17398575. We believe that different rs1129293 and rs17398575 genotypes may relate to different PI3Kγ expression, and therefore, lead to different degrees of platelet aggregation, which is determinant factor for ischemic events.^[32–34]^ However, we did not have the opportunity to investigate the possible links and mechanisms between the association of genotypes rs1129293 and rs17398575 and how they relate to PI3Kγ expression. The aforementioned aspect should be studied in future.

In addition to gene polymorphism, we found that patients’ history of hypertension also affects their responsiveness to clopidogrel. Hypertension is widely proven to be a potential cause for a reduced antiplatelet effect of clopidogrel and aspirin.^[35]^ Previous studies have identified pathophysiological links between PI3Kγ and blood pressure regulation.^[36,37]^ Carnevale et al suggest that PI3Kγ in hypertension may be a novel therapeutic target that controls vascular myogenic tone and target organ damage.^[38]^ However, in contrast to our study, a previous study also demonstrates that statin usage, smoking, overweight, diabetes, age, and female sex are all associated with the efficacy and good responsiveness to clopidogrel.^[35]^ A possible explanation for this relationship could be attributed to the fact that the platelet responsiveness value was not calculated from the difference between pre- and post-clopidogrel therapy. Therefore, the associations between clinic date and platelet responsiveness may be undercalculated. Our limitation in this study is due to our lack of direct evidence to support the association between *PIK3CG* polymorphism and the antiplatelet responsiveness of clopidogrel to use for the measurement of plasma clopidogrel concentrations. In conclusion, our study revealed that patients with TT genotype of SNP rs1129293 and GG genotype of SNP rs17398575 showed a poorer prognosis compared with patients with other genotypes. The SNP rs1129293 is proven to be associated with decreased high-density-lipoproptein cholesterol.^[39]^ Additionally, a large number of studies showed high-density-lipoproptein cholesterol to be a significant, strong, and independent inverse predictor of cardiovascular risk, and is correlated with the occurrence, development, and prognosis of CHD.^[40–42]^

In summary, our study has highlighted that *PIK3CG* SNPs (rs1129293 and rs17398575) may be associated with the interindividual variability of responsiveness to clopidogrel, whereas rs1129293 T and rs17398575 G alleles are considered to be risk factors for the onset of CHD. Moreover, *PIK3CG* SNPs (rs1129293 and rs17398575) may be also related to the incidence of ischemic events within 6 months. With current development of many drugs used to treat CHD, recent evidence indicates that the ticagrelor provides a faster, greater, and more consistent platelet inhibition activity in comparison with clopidogrel.^[43]^ Thus, a possible direction to be headed in following investigations is the comparison between the drug responsiveness of clopidogrel and ticagrelor.

Clopidogrel remains common and widely used to treat patients who suffer from CHD, especially for those who are financially challenged. Current alternative forms of treatment are either too expensive for the regimen to be maintained or have other consequences, such as ticagrelor. We believe that our study shows a favorable clinical net benefit for the treatment of patients suffering from CHD, and hope that the screening process of genotypes will be useful for future clinical implications. With our clinical data, we think that more experiments need to be conducted to further validate our results, and confirm the aforementioned associations.

## Acknowledgments

We would like to give our sincere appreciation to the reviewers for their helpful comments on this article.
